# Supplementary figures and images for: Curative treatment incorporating subjective decisions on age and frailty is not beneficial for older patients with oral cavity squamous cell carcinoma
Source: PLoS One. 2025 Aug 25;20(8):e0330376. doi: 10.1371/journal.pone.0330376 (PMC12377585; doi:10.1371/journal.pone.0330376)

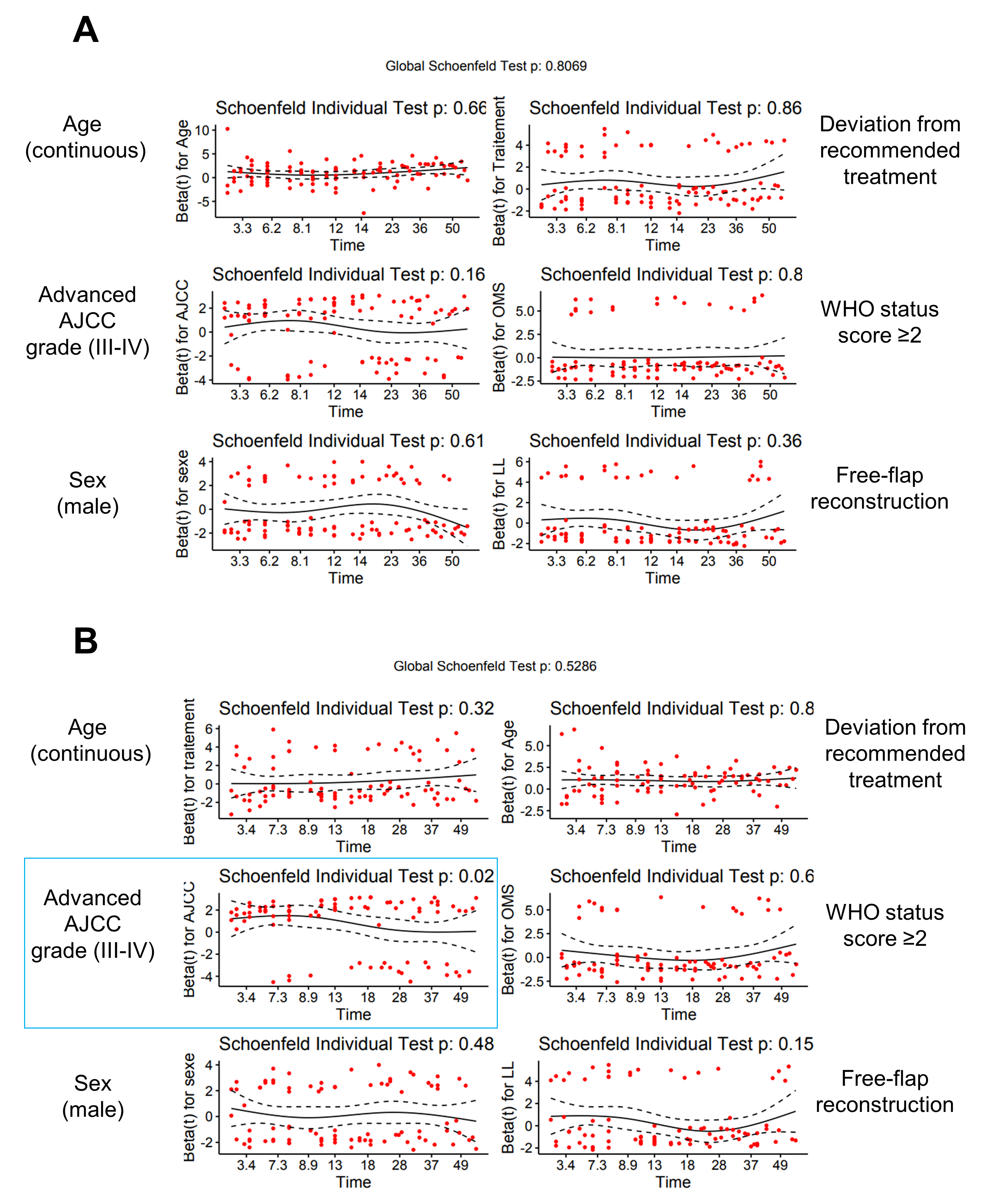

Supplement: S1 Fig — The proportional hazard assumptions were tested for the recurrence (a) and mortality (b) models with age as a continuous variable. (TIF) [file pone.0330376.s002.tif]
