# Supplementary material for: Curative treatment incorporating subjective decisions on age and frailty is not beneficial for older patients with oral cavity squamous cell carcinoma
Source: PLoS One. 2025 Aug 25;20(8):e0330376. doi: 10.1371/journal.pone.0330376 (PMC12377585; doi:10.1371/journal.pone.0330376)
Supplement: S1 Table — Modelled according to each individual WHO performance status score. (DOCX) [file pone.0330376.s003.docx]

**Supplementary Table 1.** Bivariate analyses for 3-month postoperative morbidity modelled according to each individual WHO performance status score

| **Independent variable** | **Patients, n (%)** | **3-month post-operative morbidity** | |
| --- | --- | --- | --- |
|  |  | Bivariate | |
|  |  | OR[95%CI] | p value |
| WHO performance status score (reference score=0 (n=64; 35% patients)) |  |  |  |
| 1 | 91 (49%) | 1.40[0.62-2.74] | 0.43 |
| 2 | 27 (15%) | 1.8[0.73-4.6] | 0.25 |
| 3 | 3 (2%) | NA | ≈ 1 |

OR: odds ratio
