# Supplementary material for: Curative treatment incorporating subjective decisions on age and frailty is not beneficial for older patients with oral cavity squamous cell carcinoma
Source: PLoS One. 2025 Aug 25;20(8):e0330376. doi: 10.1371/journal.pone.0330376 (PMC12377585; doi:10.1371/journal.pone.0330376)
Supplement: S2 Table — Modelled according to each individual AJCC tumor classification grade. (DOCX) [file pone.0330376.s004.docx]

**Supplementary Table 2.** Bivariate analyses for 3-month postoperative morbidity modelled according to each individual AJCC tumor classification grade

| **Independent variable** | **Patients, n (%)** | **3-month post-operative morbidity** | |
| --- | --- | --- | --- |
|  |  | Bivariate | |
|  |  | OR[95%CI] | p value |
| AJCC tumor grade (reference grade=I (n=34; 18% patients)) |  |  |  |
| II | 33 (17%) | 0.13[0.01-1.2] | 0.07 |
| III | 10 (5%) | 0.41[0.04-4.1] | 0.45 |
| IV | 108 (58%) | 3.32[1.34-8.81] | **0.01** |

OR: odds ratio
