# Supplementary material for: Curative treatment incorporating subjective decisions on age and frailty is not beneficial for older patients with oral cavity squamous cell carcinoma
Source: PLoS One. 2025 Aug 25;20(8):e0330376. doi: 10.1371/journal.pone.0330376 (PMC12377585; doi:10.1371/journal.pone.0330376)
Supplement: S3 Table — (DOCX) [file pone.0330376.s005.docx]

**Supplementary Table 3. Exploratory multivariate Cox models not adjusted for age or WHO performance status score**

| **Independent variables** | **5-year recurrence** | |
| --- | --- | --- |
|  | HR_adj_[95%CI] | p value |
| Deviation from recommended treatment | 1.9 [1.3-3.0] | **0.003** |
| Age | - | - |
| Sex (male) | - | - |
| WHO performance status score ≥2 | - | - |
| AJCC tumor classification III-IV | 1.5[1.0-2.4] | **0.05** |
| Free-flap reconstruction | - | - |
| **Independent variables** | **5-year mortality** | |
|  | HR_adj_[95%CI] | p value |
| Deviation from recommended treatment | 1.6[1.0-2.6] | **0.039** |
| Age | - | - |
| Sex (male) | - | - |
| WHO performance status score ≥2 | - | - |
| AJCC tumor classification III-IV | NA | NA |
| Free-flap reconstruction | 1.2[0.7-1.9] | 0.53 |

HR_adj_: adjusted Cox Proportional hazard ratio
