# Supplementary material for: Curative treatment incorporating subjective decisions on age and frailty is not beneficial for older patients with oral cavity squamous cell carcinoma
Source: PLoS One. 2025 Aug 25;20(8):e0330376. doi: 10.1371/journal.pone.0330376 (PMC12377585; doi:10.1371/journal.pone.0330376)
Supplement: S4 Table — (DOCX) [file pone.0330376.s006.docx]

**Supplementary Table 4. Multivariate model for deviation from recommended treatment**

| **Independent variables** | **Deviation from recommended treatment** | |
| --- | --- | --- |
|  | Multivariate | |
|  | OR_adj_[95%CI] | p value |
| Age (continuous) | 1.10[1.02-1.22] | **0.01** |
| Sex (male) | 0.50[-1.52-0.22] | 0.16 |
| WHO performance status score ≥2 | 1.10[0.10-1.92] | **0.02** |
| AJCC tumor stage III-IV | 0.63[-0.18-1.5] | 0.13 |

OR_adj_: adjusted odds ratio
