# Supplementary material for: Curative treatment incorporating subjective decisions on age and frailty is not beneficial for older patients with oral cavity squamous cell carcinoma
Source: PLoS One. 2025 Aug 25;20(8):e0330376. doi: 10.1371/journal.pone.0330376 (PMC12377585; doi:10.1371/journal.pone.0330376)
Supplement: S5 Table — (DOCX) [file pone.0330376.s007.docx]

**Supplementary Table 5. Multivariate model for free-flap reconstruction**

| **Independent variables** | **Free-flap reconstruction** | |
| --- | --- | --- |
|  | Multivariate | |
|  | OR_adj_[95%CI] | p value |
| Age (continuous) | 0.93[0.87-1.00] | **0.04** |
| Sex (male) | 1.16[0.54-2.55] | 0.12 |
| WHO performance status score ≥2 | 0.53[0.14-1.53] | 0.22 |
| AJCC tumor stage III-IV | 2.70[1.17-6.88] | **0.03** |

OR_adj_: adjusted odds ratio
