# Supplementary material for: Curative treatment incorporating subjective decisions on age and frailty is not beneficial for older patients with oral cavity squamous cell carcinoma
Source: PLoS One. 2025 Aug 25;20(8):e0330376. doi: 10.1371/journal.pone.0330376 (PMC12377585; doi:10.1371/journal.pone.0330376)
Supplement: Attachment — Submitted filename: Reponses-to-reviewers-10072025.docx [file pone.0330376.s008.docx]

Dear Dr Ubai Alsharif (Reviewer 1), Reviewer 2, and Dr John Minh,

We are grateful for your interest in our study entitled "Curative treatment incorporating subjective decisions on age and frailty is not beneficial for older patients with oral cavity squamous cell carcinoma" (Manuscript ID PONE-D-25-01028).

We believe we have addressed all the concerns raised by the Reviewers below in blue.

The newly uploaded manuscript has also been revised accordingly, with a version showing the changes made (with track changes) and a clean version (without track changes). *Note that references to page and line numbers below refer to those in the clean manuscript version.*

For the sake of clarity for both Reviewers, we wish to highlight that upon replying to the Reviewer 2 comment 2. on missing data, we realized that we had misclassified some patients with recurrence and mortality within 5 years. We have therefore reclassified all our patients and redone all statistical analyses using the R studio software. The according changes to the results have been made throughout the entire manuscript.

Equally, changes were made to our statistical models, and thus some main results, following our response to Reviewer 1 comment 13.

Reviewers' comments:

Reviewer's Responses to Questions

**Comments to the Author**

1. Is the manuscript technically sound, and do the data support the conclusions?

The manuscript must describe a technically sound piece of scientific research with data that supports the conclusions. Experiments must have been conducted rigorously, with appropriate controls, replication, and sample sizes. The conclusions must be drawn appropriately based on the data presented.

Reviewer #1: Partly

Reviewer #2: Partly

Upon answering the Reviewer comments further below, we have performed more analyses that confirm model power and lack of overfitting, thus strengthening our main findings. These new analyses and results can be found in our new manuscript.

2. Has the statistical analysis been performed appropriately and rigorously?

Reviewer #1: Yes

Reviewer #2: No

Same as above.

3. Have the authors made all data underlying the findings in their manuscript fully available?

The [PLOS Data policy](http://track.editorialmanager.com/CL0/http:%2F%2Fwww.plosone.org%2Fstatic%2Fpolicies.action%23sharing/1/010f01970b8eaf99-51b62eb5-f307-43e1-96ef-5d15cc9e0b36-000000/5QbYyiLWfuVAadnzff1t7sw-IU_NMzqZmoLkwLwWaKY=212) requires authors to make all data underlying the findings described in their manuscript fully available without restriction, with rare exception (please refer to the Data Availability Statement in the manuscript PDF file). The data should be provided as part of the manuscript or its supporting information, or deposited to a public repository. For example, in addition to summary statistics, the data points behind means, medians and variance measures should be available. If there are restrictions on publicly sharing data—e.g. participant privacy or use of data from a third party—those must be specified.

Reviewer #1: No

Reviewer #2: Yes

We have removed all mention of "data not shown" from our manuscript and now all results can be referred to either in the main text or in the Supplementary Results. We have additionally provided an anonymised database of all relevant patient demographic and clinical characteristics as Additional Materials. We have thus changed the data availability statement accordingly.

4. Is the manuscript presented in an intelligible fashion and written in standard English?

PLOS ONE does not copyedit accepted manuscripts, so the language in submitted articles must be clear, correct, and unambiguous. Any typographical or grammatical errors should be corrected at revision, so please note any specific errors here.

Reviewer #1: Yes

Reviewer #2: Yes

We thank you for your positive comments.

5. Review Comments to the Author

**Reviewer #1**:

Dear authors,

I have gladly reviewed your manuscript on this important topic. Please find my comments attached:

Kind Regards,
Ubai Alsharif

We wish to thank Dr Alsharif for reviewing our study and his constructive comments.

General issues for the whole manuscript:

1. Please point that the official guidelines are the French national guidelines in the abstract and in the methods as well.

We have made this clearer in the Abstract (page 2 line 29) and Methods (page 6 line 141), but also at the beginning of both the Results (page 10 line 214) and Discussion (page 17 line 345) sections to reinforce the context for the reader.

2. I think the terms "standard" and "non-standard" treatments should be replaced with another term such as "MDTB recommended treatment" or "Adherence to treatment recommendation" vs. "non adherence". Also, I don' think the term need to be abbreviated.

We suggest "recommended treatment" versus "deviation from recommended treatment". We prefer not to opt for a term mentioning "adherence" to avoid misinterpretations about why recommendations were not followed (i.e. there was indeed a small minority of patient refusals; refer to Figure 1).

3. please replace the term "lymph node removal" with "neck dissection" in the manuscript and in the figures.

This has been corrected throughout the manuscript.

4. The word "preoperative" is written as one word.

We thank Dr Alsharif for pointing this out. It is also the case for postoperative. All terms have been corrected throughout the manuscript and figures.

5. I am not aware of a perioperative neck dissection. Neck dissection is usually done in the same operation with tumor resection.

We have removed the misleading notion of perioperative dissection. For example, we now state in Figure 1 "19 patients did not undergo neck dissection as recommended…"

6. The manuscript is well written. However, there are minor grammatical errors and the flow of the text can be improved. If possible, it would help to revise the text once.

The logical flow, and English language and grammar of our new manuscript has been extensively corrected by one of the authors who is native English (J Butterworth).

Abstract:
7.	please rephrase the sentence "we found surgical ..." to "We included 185 patients who underwent surgical resection of OCSCC".

We have changed this sentence (page 2 line 36).
8.	Cox regression is not used to calculate the 5-year mortality or recurrence risk. The probability of death is estimated by using Kaplan-meier Method for overall survival. For recurrence, cumulative incidence function should be used. Cox regression is used to estimate hazards ratios of certain variables.

We thank Dr Alsharif for indicating our inaccurate description. We have now changed this in the Abstract for (page 2 line 34): Cox Proportional Hazards Regression was used to assess the association between NS treatment and the hazard of recurrence and mortality within 5 years. We now also state cumulative incidence rates in the Results section (page 15 line 307 and page 16 line 327).
9.	The sentence "with a tendency for a higher risk of 5-years mortality" is wrong. In a cox regression, the HR reflects an association between a variables S/NS with an outcome "Mortality" after adjustment. It has nothing to do with duration "5 years", and there are no tendencies with such a wide CI.
We have now reworded the description of these results in the Abstract (page 2 line 44), Results section (page 16 line 335), and throughout the Discussion.
10.	Line 75: A doctor is a person with doctoral degree. The authors mean "Medical practitioner or physician".
We have changed this for "physician".
Methods:
11.	I urge the authors to redo the statistical analysis while stratifying WHO performance status score by point 1,2 and more than two, and AJCC tumor stage into I, II, III, and IV. This would allow for a better comparison of results for future studies.

We appreciate your comment and have repeated the statistical analyses accordingly. The results and their interpretations can be found below:

To begin, here are the numbers of patients with each WHO performance status score (multicategorical variable):

Score 0 n=64

Score 1 n=91

Score 2 n=27

Score 3 n=3

Here are the bivariate odds ratios (OR) per individual score for 3-month post-operative morbidity, each compared to the reference score of 0:

WHO score 1: OR = 1.33 → not significant (p = 0.43)

WHO score 2: OR = 1.76 → not significant (p = 0.25)

WHO score 3: extremely low OR, highly likely due to the extremely low number of patients in this category → p ≈ 1

These results are summarized here:

| **Independent variables** | **3-month post-operative morbidity** | |
| --- | --- | --- |
|  | Bivariate | |
|  | OR[95%CI] | p value |
| **WHO performance status score (ref =0)** |  |  |
| **1** | 1.4[0.6-2.7] | 0.43 |
| **2** | 1.80.7-4.6] | 0.25 |
| **3** | NA | ≈ 1 |

In addition, WHO performance status scores are often clinically grouped as 0 and 1 —fully ambulatory with no/mild symptoms — or 2 and 3 — restricted ambulatory with moderate symptoms [1]. Overall, we therefore consider it more relevant to leave WHO score as a binarized variable in our models: score 1 versus scores≥2.

Next, here are the numbers of patients with each AJCC grade (multicategorical variable):

I n=34

II n=33

III n=10 (relatively low)

IV n=108

Here are the bivariate odds ratios (OR) per individual grade for 3-month post-operative morbidity, each compared to the reference grade of I:

|  | **3-month post-operative morbidity** | |
| --- | --- | --- |
|  | Bivariate | |
|  | OR[95%CI] | p value |
| **AJCC classification grade (ref AJCC grade I)** |  |  |
| **II** | 0.13[0.01-1.2] | 0.07 |
| **III** | 0.41[0.04-4.1] | 0.45 |
| **IV** | 3.32[1.3-8.8] | **0.01** |

Only AJCC stage IV was found positively associated with 3-month post-operative morbidity.

Clinically, stages I and II correspond to localised or early-stage cancers, with a better prognosis. Stages III and IV refer to more advanced cancers, with regional involvement (stage III) or distant metastases (stage IV), and therefore have poorer prognoses. Early stages are often treated with curative intent (surgery, localised radiotherapy). Advanced stages generally require systemic (chemotherapy, immunotherapy) or palliative approaches [2, 3]. Overall, given both the small number of patients with AJCC stage III, and the clinical relevance of binarizing this variable, we wish to leave AJCC stage grouped as I-II versus III-IV in our models.

Finally, the "age" variable has now been incorporated into models as a continuous rather than a categorical variable (≥80 years) (page 9 line 190). The linear effect for 3-month post-operative morbidity is not significant either: bivariate odds ratio of 0.97[0.9-1.03]; p=0.29

We have now updated the regression results in Table 4 with age as a continuous variable, with age showing a significant independent association with 5-year mortality.

The bivariate analyses for each individual WHO score and AJCC tumor stage can now be found in Supplementary, as cited in the main manuscript (page 13 line 265).

12.	The adherence to treatment is probably associated with age. We should question whether it's correct to include age and treatment as variables in the cox regression.

We thank Dr Alsharif for this relevant comment. We checked the potential association between age and treatment in our data. The Pearson correlation coefficient between age and treatment is 0.40, indicating a moderate correlation. However, this level of correlation does not reveal any problematic collinearity for the joint inclusion of these variables in the Cox regression models. Indeed, the variance inflation factor of 1.01 remains below the generally accepted threshold of 5. Furthermore, age and treatment are two distinct variables of clinical interest that are likely to independently influence survival. Excluding them from the model could lead to confounding biases. We therefore believe it is justified to retain these two variables in our multivariate Cox models. Nevertheless, we have added a sentence to the Methods section specifying that multicollinearity was verified (page 9 line 202).

13.	The proportional hazards assumption needs to be tested for age as categorical variables, and it would not hold probably. If this is the case, then the cox regression has to be stratified by age as well. Only this would show the true HR for treatment especially in the overall survival model. I urge the authors to re-run the statistical analysis after considering my recommendation in the results section as well.

Accordingly, we have checked that the effect of age, in the different classes of <75, 75–85, >85 years, on the risk of recurrence and mortality remains constant over time; this is a required condition for the correct use of a Cox model (proportional hazards model).

|  | Df | **Recurrence model** | | **Mortality model** | |
| --- | --- | --- | --- | --- | --- |
|  |  | Chisq | P value | Chisq | P value |
| Age | 2 | 0.7 | 0.70 | 0.9 | 0.63 |

Below are the Schoenfeld residual plots testing the assumption of proportional hazards for stratified age.

Recurrence:

Mortality:

The proportional hazards assumption was then tested for each variable included in the Cox models using Schonenfel residual analysis. These results are presented in the table below. Age was thus included in Cox models as a continuous variable.

|  | Df | **Recurrence model** | | **Mortality model** | |
| --- | --- | --- | --- | --- | --- |
|  |  | Chi-squared | P value | Chi-squared | P value |
| Non-standard treatment | 1 | 0.02 | 0.86 | 0.95 | 0.32 |
| Age (continuous) | 22 | 18.7 | 0.66 | 15.7 | 0.82 |
| AJCC grade III-IV | 1 | 1.93 | 0.16 | 5.21 | **0.02** |
| WHO score≥2 | 1 | 0.04 | 0.83 | 0.2 | 0.65 |
| Sex (male) | 1 | 0.24 | 0.62 | 0.48 | 0.49 |
| Reconstruction performed | 1 | 0.8 | 0.37 | 1.98 | 0.16 |
| **Global** | **7** | **20.55** | **0.81** | **25.82** | **0.52** |

Recurrence model:

The residuals are scattered randomly around zero with no visible trend over time. All p values were >0.05. We can thus confirm that assumptions are met for our Cox proportional hazard model for recurrence (page 15 line 315).

Mortality:

Including age as a continuous variable in the mortality model resulted in a significant violation of the proportional hazards assumption for the AJCC variable (χ² = 5.22, p = 0.022), while all other covariables complied with this assumption. We therefore reconstructed this model by stratifying with AJCC stage to ensure the stability of the effect estimates (page 16 line 328).

14.	The authors should keep in mind that there is confounding by indication in this study. Patients who did not adhere to a certain therapy, probably did not adhere in the first place because of a worse general health condition or a higher age. The adherence in itself is a result of age and general condition. So is incorporating age and general condition into the statistical models correct in the beginning? This might be worth to incorporate as an additional model in the appendix.

Propensity scoring or an inverse probability weighting model would have been appropriate to limit the indication bias inherent in our observational study. However, due to our relatively small cohort size, this approach was not chosen. Instead, we opted for classic multivariate models supplemented by sensitivity analyses (below and Supplementary Table 3). Age and WHO score were not adjusted for in these exploratory multivariate models.

| **Independent variables** | **-5-year recurrence** | |
| --- | --- | --- |
|  | Sensitivity | |
|  | HR_adj_[95%CI] | p |
| **Non-standard treatment** | 1.9 [1.3-3.0] | 0.003 |
| **Age** | ~~1.03 [1.00-1.10]~~ | - |
| **Sex (male)** | - | - |
| **WHO performance status score ≥2** | - | - |
| **AJCC tumor classification III-IV** | 1.5[1.0-2.4] | 0.05 |
| **Free-flap reconstruction** | - | - |
|  | **5-year mortality** | |
| **Independent variables** | Sensitivity | |
|  | HR_adj_[95%CI] | p |
| **Non-standard treatment** | 1.63[1.02-2.6] | 0.039 |
| **Age** | ~~1.05 [1.00-1.09]~~ | - |
| **Sex (male)** | - | - |
| **WHO performance status score ≥2** | - | - |
| **AJCC tumor classification III-IV** | NA | NA |
| **Free-flap reconstruction** | 1.17[0.71-1.93] | 0.53 |

Non-standard treatment was found significantly associated with an increased risk of recurrence and mortality within 5 years. This suggests that the outcomes of non-standard treatment cannot be explained solely by age or WHO score (general condition).

Nonetheless, we acknowledge the existence of indication bias in our study, in which treatment allocation is influenced by patient age and general condition (see additional multivariate analysis below and Supplementary Table 4). Treatment is not randomised (page 19 >line 398); it depends on age and general condition, which are themselves linked to prognosis (survival, recurrence). However, we want to know whether non-standard treatment increases the risk of recurrence or mortality, regardless of the effects of age and WHO status.

| **Independent variables** | **Non-standard treatment** | |
| --- | --- | --- |
|  | Multivariate | |
|  | OR_adj_[95%CI] | p value |
| **Age** | 1.1[1.02-1.2] | **0.01** |
| **Sex (male)** | 0.5[-1.5-0.22] | 0.16 |
| **WHO performance status score ≥2** | 1.1[0.1-1.9] | **0.02** |
| **AJCC tumor classification III-IV** | 0.6[-0.18-1.5] | 0.13 |

Results:

15.	Please state why the 19 patients did not undergo neck dissection. Did they refuse or was it the surgeon?

No study participants refused surgery. Following decisions made in preoperative multidisciplinary tumor boards, n=19/185(10%) patients did not undergo neck dissection as initially recommended by the French guidelines (page 10 line 216). These subjective decisions were based on adapting treatment to patient age and frailty (page 10 line 217). Please also refer to the Discussion (page 18 line 373) where we further explore variables associated with the indication of non-standard treatment.

16.	There is a redundancy in the paragraph of the lines 187-194. I think it can be simplified with a referral to figure 1.

Accordingly, we have lightened the text where we refer to Figure 1 (page 10 lines 215– 227).

17.	Table 2: Please split Table 2 into 3 columns NS group, S group and overall, and report all variables stratified by treatment. In addition, please put the outcome measures after the independent variables.

We have made these worthwhile changes.

18.	In possible, please list the WHO performance status score by point 1,2 and more than two, and AJCC tumor stage into I, II, III, and IV.

We do not feel this necessary in the main text, where we proceed with analyses grouping WHO scores and AJCC tumor stage. These data can however be found in Supplementary, as cited in the main manuscript (page 13 line 265).

19.	if available report the histological criteria associated with poor prognosis in your group in Table 2.

We do not consider it relevant to add specific histological criteria to Table 2. Instead, we have indicated in the caption to Figure 1 (page 27 line 620) that patients were recommended adjuvant treatment according to postoperative (laboratory) TNM classification (>pT2, presence of adenopathy) and histological criteria associated with poor prognosis (<1mm margins, lymphatic emboli, perineural infiltration). A total of n=123/185(66%) patients were recommended adjuvant treatment according to these guidelines, among which 86% received this treatment.

20.	Table 3: Please stratify here also by treatment group. I would add the outcome measures from Table 2 to Table 3.

We have now stratified the data according to treatment type. However, we wish to retain the outcome measures in Table 2 where we also evoke the independent variables investigated; i.e. the study design. Table 3 describes the specific complications within 3 months.

21.	The sentence "Indeed, .." in lines 246-248 is wrong. This is not the interpretation of HR of 1.6. Please correct.

We thank Dr Alsharif for indicating our misinterpretation. The result has since changed and we have corrected our interpretation (page 13 line 277).

22.	There is no need for the new titles on the liens 250-251 and 258-259 and 273-274. Consider using one subtitle for all there paragraphs.

We agree that we can regroup results from the multivariate analyses in Table 4 under this single title: Deviation from recommended treatment is independently associated with increased 3-month postoperative morbidity and recurrence within 5 years

23.	There is no need to report two digits after the comma in the CI when it's higher than 10 as in Line 262.
We have harmonized CI reporting throughout the entire manuscript.

Discussion:
24.	The sentence in the lines 298-299 is not true. There is no tendency.

These data have now changed since our new analyses, but we indeed now more accurately state that deviation-from-guidelines treatment is not associated with increased risk of mortality (page 16 line 335 and Table 4).

25.	the statement in the lines 329 - 331. Well as I mentioned before, there is a reason why the patients did not receive the full treatment and it's probably the reason for the postoperative morbidity. It is probably the general condition.

We appreciate that the only way to accurately decipher the effect of standard versus non-standard treatment on our outcomes is by performing a randomized trial comparing patients receiving and not receiving treatment according to the official guidelines (page 19 line 400). We have now better adapted this section of the Discussion to explain how and why older age and worse WHO scores were associated with deviation from recommended treatment (Supplementary Table 4).

Figure 1:
26.	I would suggest improving the figure a bit, by adding two blocks that show preoperative and postoperative MDTB. In addition, I suggest using the terms adhered to the postoperative MDTB recommendation.

We have added colour to make the difference between pre and postoperative MDT boards more explicit. The new description can also be found in the new Figure caption (page 27 line 618).

**Reviewer #2**:

1. I believe the argument for the study could be made more forcefully. For example: "Although clinicians often deviate from standard treatment guidelines when managing older patients with OCSCC—relying instead on subjective judgments of age and frailty—the impact of these real-world, non-standard decisions on patient outcomes remains poorly understood."

We thank reviewer 2 for their reading and insightful comments on our study.

We have now reinforced the arguments for performing our study in the Abstract (page 2 >line 26) and Introduction (page 5 line 112).

2.	In the methods section, the authors need a clear account of how they handled missing data. This is critical in retrospective studies.

We report no missing baseline data or data on complications within 3 months after surgery; patients required a heavy multidisciplinary management and so all data were found during hospitalization and short post-operative follow-up.

Patients then lost to follow-up during the study period were censored and analysed. We have made this clearer in the Methods section (page 8 line 185 and page 9 line 199).

Upon checking these data, we realized that we had misclassified some patients in the recurrence (notably) and mortality within 5 year groups. We have therefore reclassified all our patients and the new data has slightly changed for: recurrence within 5 years was recorded for 101 (not 108) patients (84 censored patients, including 46 patients lost to follow-up). Mortality was recorded for 88 patients (not 87) (97 censored patients, including 39 lost to follow-up). Please refer to Table 2 and page 10 line >231.

Kaplan-Meier plots have been reconstructed accordingly for recurrence-free survival (Fig 2a page 15 line 307) and overall survival (Fig 2 b page 16 line 326):

3.	Also, the methods section and the results section could use a little reorganization.

Accordingly, we have broken the text down into more sections in the Methods and regrouped multivariate regression results in the Results section (as also requested by Reviewer 1). The Results section has also been condensed, with many sentences being reformulated to more accurately interpret our findings.

4.	In the methods section, the authors should clearly state the number of outcome events expected, justify the number of predictors retained per model, and acknowledge the risk of overfitting, especially for the morbidity model. In the discussion, the authors should reflect on the model power, especially the weak mortality result (HR 1.3, CI includes 1, p=0.31).

It is recommended to have at least n=10 events per variable in regression models, including for independent variables in Cox models [4]. For the multivariate model assessing post-operative morbidity (Clavien-Dindo grade ≥III), 51 patients developed morbidity among 184 patients. The final model included three predictors: treatment type, advanced AJCC score, and with/without free-flap reconstruction (refer also to Reviewer 1 comment 11 above), with significant associations in univariate analyses (Table 4). The event/variable ratio (EVR) was 17 (page 15 line 301), which is well above the commonly accepted cutoff of 10, thus the likelihood of overfitting is minimized.

Likewise, the risk of overfitting was also taken into account for the Cox models. The number of events observed were 88 mortalities and 101 recurrences. The 5-year recurrence model included 3 variables (EVR = 33.7 page 16 line 317), and the 5-year mortality model included 4 variables (EVR = 22 page 16 line 332). In both cases, the event/variable ratio was greater than the recommended threshold of 10, ensuring estimate robustness.

We have now changed our interpretation of the non-significant mortality results (page 16 line 335).

5.	Furthermore, the authors MUST address the assumptions for the inferential statistics. Were all the assumptions met for the Cox proportional hazard and logistic regression? If all assumptions were met, a global statement would suffice. If not, then the authors should explain why they proceeded with the study.

The proportional hazards assumption has now been tested for each variable included in the Cox models. Please refer to Reviewer 1, remark 13 (page 5 above).

6.	In the results, there is no discussion of statistical power, nor is there a post-hoc power analysis. So, it is not clear if the study had sufficient power to detect the observed effects. Lastly, the risk of overfitting in the morbidity model is not adequately discussed, considering the relatively small number of events. This could undermine the reliability of the model’s findings.

We feel that our aforementioned new analyses performed (a posteriori proportional hazards assumption testing and event/variable ratio calculations), with their results mentioned throughout the Results section, allow us to confirm minimal risk of model overfitting. We have additionally changed our interpretation of the association between deviation from recommended treatment and mortality within 5 years to non-significant throughout the manuscript; i.e. no effect.

7.	Overall, I found the manuscript well-written with only minor stylistic errors. I hope to see a revised draft soon.

The logical flow and structure, as well as the English language and grammar, of our new manuscript has been extensively corrected by one of the authors who is native English (J Butterworth).

Again, we would like to thank the Reviewers for their comments. We hope that our responses and revised version of our manuscript are clearer and strengthen our research. Please do not hesitate to contact me if you require further clarification.

Yours sincerely,

Alice Prevost (MD, PhD)

**REFERENCES**

1.	Yang M-H, Chen T-H, Wang H-M, et al (2024) Prognostic factors and risk-stratification model of recurrent or metastatic head and neck squamous cell carcinoma treated with cetuximab containing regimen. BMC Cancer 24:1227. https://doi.org/10.1186/s12885-024-12425-0

2.	Zanoni DK, Patel SG, Shah JP (2019) Changes in the 8th Edition of the American Joint Committee on Cancer (AJCC) Staging of Head and Neck Cancer: Rationale and Implications. Curr Oncol Rep 21:52. https://doi.org/10.1007/s11912-019-0799-x

3.	Kadoya K, Yagihara K, Katsurano M, et al (2025) Prognosis in elderly head and neck cancer patients without indication for treatment. Oral Sci Int 22:e1271. https://doi.org/10.1002/osi2.1271

4.	Peduzzi P, Concato J, Feinstein AR, Holford TR (1995) Importance of events per independent variable in proportional hazards regression analysis. II. Accuracy and precision of regression estimates. J Clin Epidemiol 48:1503–1510. https://doi.org/10.1016/0895-4356(95)00048-8
